# Inferring Developmental Stage Composition from Gene Expression in Human Malaria

**DOI:** 10.1371/journal.pcbi.1003392

**Published:** 2013-12-12

**Authors:** Regina Joice, Vagheesh Narasimhan, Jacqui Montgomery, Amar Bir Sidhu, Keunyoung Oh, Evan Meyer, Willythssa Pierre-Louis, Karl Seydel, Danny Milner, Kim Williamson, Roger Wiegand, Daouda Ndiaye, Johanna Daily, Dyann Wirth, Terrie Taylor, Curtis Huttenhower, Matthias Marti

**Affiliations:** 1 Department of Immunology and Infectious Diseases, Harvard School of Public Health, Boston, Massachusetts, United States of America; 2 Department of Biostatistics, Harvard School of Public Health, Boston, Massachusetts, United States of America; 3 Malawi-Liverpool-Wellcome Trust Clinical Research Programme, Blantyre, Malawi; 4 Liverpool School of Tropical Medicine, Liverpool, United Kingdom; 5 The Broad Institute of Harvard and MIT, Cambridge, Massachusetts, United States of America; 6 College of Osteopathic Medicine, Michigan State University, East Lansing, Michigan, United States of America; 7 Blantyre Malaria Project, University of Malawi College of Medicine, Blantyre, Malawi; 8 Department of Pathology, Brigham and Women's Hospital, Boston, Massachusetts, United States of America; 9 Department of Biology, Loyola University Chicago, Chicago, Illinois, United States of America; 10 Faculty of Medicine and Pharmacy, Cheikh Anta Diop University, Dakar, Senegal; 11 Department of Medicine, Albert Einstein College of Medicine, Bronx, New York, New York, United States of America; National Center for Biotechnology Information (NCBI), United States of America

## Abstract

In the current era of malaria eradication, reducing transmission is critical. Assessment of transmissibility requires tools that can accurately identify the various developmental stages of the malaria parasite, particularly those required for transmission (sexual stages). Here, we present a method for estimating relative amounts of *Plasmodium falciparum* asexual and sexual stages from gene expression measurements. These are modeled using constrained linear regression to characterize stage-specific expression profiles within mixed-stage populations. The resulting profiles were analyzed functionally by gene set enrichment analysis (GSEA), confirming differentially active pathways such as increased mitochondrial activity and lipid metabolism during sexual development. We validated model predictions both from microarrays and from quantitative RT-PCR (qRT-PCR) measurements, based on the expression of a small set of key transcriptional markers. This sufficient marker set was identified by backward selection from the whole genome as available from expression arrays, targeting one sentinel marker per stage. The model as learned can be applied to any new microarray or qRT-PCR transcriptional measurement. We illustrate its use *in vitro* in inferring changes in stage distribution following stress and drug treatment and *in vivo* in identifying immature and mature sexual stage carriers within patient cohorts. We believe this approach will be a valuable resource for staging lab and field samples alike and will have wide applicability in epidemiological studies of malaria transmission.

## Author Summary

The human malaria parasite *Plasmodium falciparum* is transmitted through a mosquito vector and causes over half a million deaths per year. The microorganism cycles through asexual and sexual life cycle stages, and its successful transmission relies on cells in the sexual stage. These stages are, however, present only at low levels during infection; most infecting cells are asexually reproduced. It can be challenging to assign biomolecular activity to particular parasite life cycle stages from typical gene expression profiles, given the mixed stage composition of most samples. We developed a deconvolution model to identify components of *Plasmodium* transcriptional activity contributed by sexual and asexual life cycle stages, initially using samples of known composition. From these, we optimized a small set of stage-specific genes with highly informative expression patterns and trained an inference model to predict the stage composition of new samples. The model successfully inferred the parasite's transition from asexual to sexual development over time under laboratory conditions and identified a subset of patient samples harboring transmissible sexual stages. The system presented here can aid in epidemiological or laboratory perturbation in which stage composition is an important step in understanding and preventing malaria transmission.

## Introduction

One of the tenets of the recently released Malaria Eradication Research Agenda (malERA) is the development of new diagnostics specifically addressing transmission reduction [1]. Individuals harboring the *Plasmodium falciparum* transmissible parasite stage, or gametocyte, are the primary reservoir for malaria transmission, and thus proper surveillance of gametocyte carriers is critical to transmission reduction. Surveillance is difficult, however, because gametocytes comprise only a small fraction of the total body parasite load during active infection and are only observed in the bloodstream in their mature form, while developing stages are sequestered in tissues [2]. For these reasons, quantifying gametocytes in mixed parasite populations has been an ongoing challenge ever since they were first identified more than a century ago.

Gametocytes do execute substantially different transcriptional programs from asexual parasite stages, however, as has been well-studied *in vitro* [3]. Like the sequential dynamics of the asexual *Plasmodium* life cycle [4], [5], gametocytes develop in a staged progression from immature (young and intermediate stages) to mature transmission-competent cells in preparation for meiosis and further development in the mosquito vector. The switch between asexual replication and sexual development does not occur ubiquitously *in vivo* or *in vitro*, as even the most synchronized gametocyte induction protocols result in partially asynchronous and mixed gametocyte stages [3], [6]. This problem is compounded *in vivo*, as blood sampled during infection is likely to contain both gametocyte and asexual parasite populations, leading to a highly convolved transcriptional mixture.

In addition to the need to dissect these signatures for analysis of microarray data, it is also of interest to develop a field-friendly approach for detecting and quantifying both immature (indication of conversion to sexual development) and mature (indication of infectiousness to mosquito vector) gametocyte stages. Transcriptional approaches such as RT-PCR, QT-NASBA and RT-LAMP have been developed [7], [8], [9] using the established mature gametocyte marker *Pfs25* and the putative immature gametocyte marker *Pfs16*. While these approaches enable sensitive detection of these transcripts, it is unclear how the detection of these transcripts - particularly *Pfs16* - relates to actual gametocyte carriage [8]. The development of a qRT-PCR-based assay has thus far been impeded primarily because this approach cannot distinguish transcript from genomic DNA when sequences are identical; the majority of *P. falciparum* genes lack introns and thus have identical sequences for both RNA and DNA. It is therefore worth identifying novel intron-containing markers for which exon-exon junction-spanning primers can be designed so that this approach can be used for *in vivo* gametocyte quantification.

Our goal was thus to develop a new transcript-based gametocyte model that addressed these challenges. Using a deconvolution approach, we quantified the stage-specificity of *Plasmodium* transcripts genome-wide and subsequently identified intron-containing markers from across the full range of asexual and sexual development. In order to identify expression patterns specific to different gametocyte stages, particularly the immature stages, existing *in vitro* asexual and sexual developmental time course samples were re-analyzed to account for their mixed stage composition. We further developed a qRT-PCR assay based on these results and, applying the model in reverse, established an algorithm to estimate the amounts of immature and mature sexual and asexual stages in a patient sample based on the expression of a small set of stage-specific markers. This was inspired by related approaches that have been used successfully in dealing with mixed cancerous/non-cancerous tissue samples [10], [11] and with mixed stages of budding yeast [12]. This framework is implemented for public use at http://huttenhower.sph.harvard.edu/malaria2013; as a transmission-focused tool, this system can be applied in epidemiological settings, and as such will ideally support efforts directed toward reducing malaria prevalence worldwide.

## Results

### A regression model characterizing genome-wide stage-specific transcript expression in *P. falciparum*

#### Calculation of gene-specific expression contributions to each life cycle stage

To identify expression patterns of individual stage categories while accounting for mixed stage composition, we began by using a labeled set of microarray data with asexual and gametocyte proportions assessed manually by microscopy [3], [5]. For our purposes, we divided the *P. falciparum* intra-erythrocytic development into categories that reflect physiologically relevant distinctions during the course of natural infection in the human host. Specifically, we separated those phases found sequestered in tissues from those found in circulation, resulting in a total of five categories. These included two asexual categories, i) the circulating ring stage (termed “R”) and ii) the sequestering trophozoite and schizont stage (termed “T”). The three gametocyte categories were i) the young (Stage I, termed “YG”) and ii) intermediate (gametocyte stages II, III, IV, termed “DG”) immature gametocytes that are absent from circulation and iii) the circulating mature stage V gametocyte (termed “MG”). For most analyses, we grouped YG and DG together as one category encompassing all immature gametocytes (termed “IG”).

**Figure 1:**
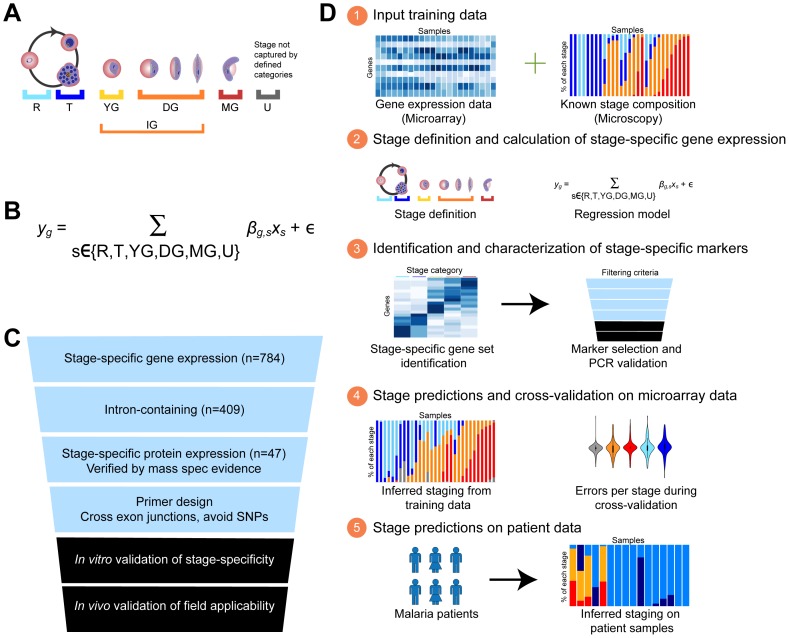
*In silico* dissection approach developing a linear regression model to identify stage-specific gene expression profiles within bulk parasite population gene expression. (A) Definition of physiologically relevant stage categories within *P. falciparum* development for which we will identify stage-specific expression signatures. Stages are as follows: R: asexual ring, T: asexual trophozoite and schizont, YG: young gametocyte ring and stage I, DG: developing gametocyte stages II, III, and IV, IG: all immature gametocytes (YG+DG), MG: mature gametocyte stage V, and U: unexpected profile not captured by our defined stages. (B) Linear regression model for the deconvolution of bulk gene expression data from mixed stage samples. Terms are as follows: *y_g_*: total expression of gene g, β*_g,s_*: expression of gene g attributed to stage s, *X_s_*: proportion of the sample that is stage s. (C) Marker Selection. Filters used to narrow down gene sets to our set of sentinel markers for field-applicable qRT-PCR assay. As we chose markers for ring and trophozoite/schizont stages *a priori* based on published stage-specific gene expression data for asexual development [4], [5], [46], we used this selection method to identify markers for the remaining gametocyte stage categories. (D) Overall stage prediction schematic.

We subsequently constructed a constrained regression model to identify the degree to which each *P. falciparum* gene's expression varied with respect to life cycle stage (Figure 1). The model parameters encode the relative expression of each gene that can be attributed to each life cycle stage, five of which are described above and a sixth category representing transcriptional activity not well-captured by any of these (Figure 1A). The model was initially fit using microarray expression data from three time courses spanning the *P. falciparum* intra-erythrocytic life cycle wherein the stage distributions at each of the time points were determined by microscopy [3], [5] (Figure 1D-1).

**Figure 2:**
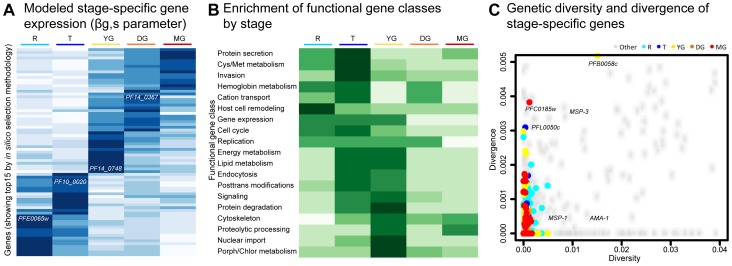
Stage-predictive gene sets are enriched for specific biological processes but show no signature of selection by diversity/divergence measures. (A) Top 15 model *β_g,s_* parameters specific to each stage; values indicate for each gene the degree of its expression attributed to each stage. (B) Gene set enrichments of GO and KEGG processes by stage (Supplementary Table S2). (C) Genetic diversity (within patient) vs. divergence (between isolate) of the *P. falciparum* genome (see Methods for data sources), highlighting genes identified as stage-specific. Several known markers are labeled for reference.

In any one time point or sample, the total transcript abundance *y_g_* for each gene *g* was modeled as a mixture of its abundance in each specific stage. The mixture fraction *x_s_* represented the fraction of parasites in stage *s* (Figure 1B), and the model was constrained to require the sum of *x_s_* across stages to remain equal to one. The contribution of each stage to *g*'s overall transcript abundance was captured as *β_g,s_* parameters which provided not only predictive accuracy but were also used to identify stage-specific gene sets and pathways (Figure 1D-2). After identifying each gene *g*'s stage-specific parameters *β_g,s_*, but before winnowing them down to a minimal set of sentinel markers, we inspected the resulting genome-wide characterization of *P. falciparum* life cycle transcriptional activity in order to identify stage-specific pathways and regulatory mechanisms.

#### Definition of stage-specific gene sets reveals parasite biology

The model parameters *β_g,s_* provided a measure of the amount of expression of each gene attributable to each stage *s*. To initially identify genes with stage-specific regulation, we selected those genes for each stage where *β_g,s_* was at least two (for sexual stages) or one (for asexual stages) standard deviations further above mean than for any other stage. This process resulted in 637 stage-specific genes distributed across five stages: 154 (R), 34 (T), 229 (YG), 34 (DG) and 186 (MG), with the top fifteen individual stage-specific markers appearing in Figure 2A (see Supplementary Table S1 for genome-wide analysis).

#### Gene set enrichment analysis

Prior to selecting individual stage-specific sentinel genes from these data, stage-specific pathway activity was assessed genome-wide using Gene Set Enrichment Analysis (GSEA). Since the standard gene sets available for GSEA are somewhat sparse for *P. falciparum*, a pathway database was constructed using the gene annotations in PlasmoDB for the Gene Ontology [13] (see also Supplementary Tables S2 and S3). These gene sets were then used with GSEA on our model parameters, employing the genome-wide *β_g,s_* parameters z-scored across stages as a pre-ranked statistic for enrichment testing. The resulting stage-specific pathway enrichments included biological activities that are known to be associated with particular stages in the asexual cycle, such as host cell remodeling in ring stages (R) and host cell invasion and replication in the later stages of the asexual cycle (T). Our analysis supports earlier observations from the initial gametocyte transcriptome study and a more recent early gametocyte proteome [3], [14] that mitochondrial and lipid metabolism are significantly up-regulated during early sexual development, as well as factors involved in cell cycle control. We also observed enrichment of endocytic pathways and cytoskeletal remodeling during later stages of sexual development. These processes are likely linked to hemoglobin uptake and exflagellation during male gametogenesis, respectively (Figure 2B, and Supplementary Table S2).

#### Signatures of natural selection

Interaction with the host immune system can result in a higher rate of single nucleotide polymorphisms (SNPs) in genes involved in these interactions. Since life cycle stages vary in their level of interaction with the host immune system, we were interested in whether any of our stage-specific gene sets varied in signatures of natural selection. As a measure for balancing selection within a parasite population, we determined the genetic diversity of individual genes within the three gametocyte stages and two asexual stages (Figure 2C). For this purpose, we calculated SNP **π** values for each gene based on a sequence comparison from 25 culture-adapted strains from Senegal [15]. SNP **π** quantifies the average number of pairwise differences among a set of strains at a set of assayed SNPs. As a complementary measure for positive selection between populations, we also calculated Fst [16] when comparing the 25 strains from Senegal with a reference line from Honduras, HB3 [17]. Our analysis demonstrated that none of the genes from within the set of highly stage-specific asexual and sexual markers are under increased selection compared to the genomic average, neither within a population nor between two populations. This suggests that the stage-specific transcripts identified by this process are enriched, by these criteria, for gene products core to the regulatory program or stage transitions themselves, excluding proteins that would interact more directly with the host. Such conserved genes are likely to serve as robust markers in lab and field samples alike.

### Selection and validation of a sentinel marker set for stage prediction

#### Marker selection overview

**Figure 3:**
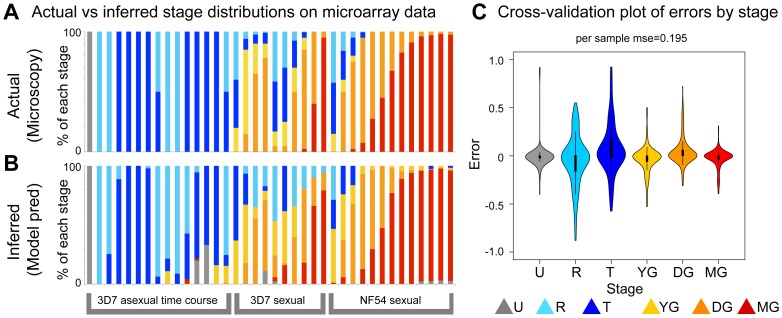
Marker selection yields a set of sentinel markers with high predictive accuracy. (A) Actual and (B) inferred stage distributions across five microarray time courses (two asexual and three sexual) with reference stage distributions determined by microscopy. Six markers were used to make these predictions, five as identified through filtering criteria (Table 1) and the previously established mature gametocyte marker *Pfs25*. (C). Bootstrap cross-validation of error rates expected per stage in model inferences. Violin plots show expected density, with internal boxplots detailing the 25^th^–75^th^ percentiles and 1.5× fences.

After characterizing the genome-wide stage-specific gene expression of *P. falciparum*, we proceeded to identify a small subset of markers sufficient to recapitulate genome-wide resolution for stage prediction. To combine predictive accuracy with biological interpretability, we chose to identify the single markers representing each stage category while still minimizing prediction error. This resulted in a set of filtering criteria to obtain markers validated to be stage-specific and suitable for use in microarray and qRT-PCR analyses (Figure 1C and 1D-3).

#### Selection of asexual-specific and constitutively expressed markers

To identify optimal markers for the better studied asexual R and T stages, we began with the stage-specific gene sets identified for each stage and first filtered based on presence of intron(s). We also required mass spectrometry evidence confirming the protein is expressed during asexual development [14], [18]. Next, we ranked genes based on frequency of retention during model predictive marker selection (see Methods) and used raw expression information such as lack of allelic expression variation, high absolute expression levels [5], and stage-specific expression evidence by expression timing [4] as additional filters. Based on this process, we selected *PFE0065w* for the early asexual stage (R). It encodes skeletal-binding protein 1 (SBP1), a well-characterized component of parasite-induced membrane structures in the host red blood cell (RBC) termed Maurer's clefts [19]. Using these same criteria, we selected *PF10_0020* as the late asexual stage (T) marker. The gene encodes a protein that is predicted to be secreted into the host RBC [20] and contains a putative alpha/beta hydrolase domain. It is highly specific for late asexual stages not only based on the β*_g,s_* parameter, but also based on its independent gene expression profile from a comparative transcriptional analysis of three *P. falciparum* strains [4]. For the baseline marker, we chose a constitutively expressed transcript as determined by ranking all *P. falciparum* genes by the lowest standard deviation across life cycle stages [3], [5] and patient isolates [21] and using a cut-off for high expression. The selected marker *PF11_0209* encodes a conserved protein of unknown function.

#### Selection of gametocyte stage-specific markers

To select gametocyte markers, we began with the stage-specific gene sets identified for each stage category as above (YG, DG, or MG) and filtered first based on the presence of intron(s). Next, we retained only those genes in which mass spectrometry data again confirmed expression in the gametocyte development and absence in asexual development, providing evidence both for stage-specific activity and lack of evidence for non-specific stages. These criteria revealed a shortlist of gametocyte-specific candidates with high predictive accuracy, but in the case of the MG stage, no markers showed high and stage-specific expression levels (as defined by the β*_g,s_* parameter) comparable to the currently used gametocyte marker *Pfs25*. We therefore decided to include *Pfs25* as a MG marker to model stage distribution in microarray data, as the major criterion against using it for qRT-PCR (i.e. absence of an intron), does not apply to the analysis of chip-based data. For the remaining gametocyte stage categories in which highly expressed markers were successfully identified, we ranked gene lists based on frequency of retention during model predictive marker selection and selected *PF14_0748* and *PF14_0367* to represent young (YG) and developing (DG) gametocytes, respectively. *PF14_0748* has previously been identified as an early gametocyte marker [3], [6], and it has been demonstrated that its promoter can drive gametocyte-specific reporter expression [6], [22]. It encodes a protein predicted to be exported into the host RBC with a putative PHIST domain [20]. *PF14_0367* has not been described previously.

#### Initial validation of the model on microarray samples

**Figure 4:**
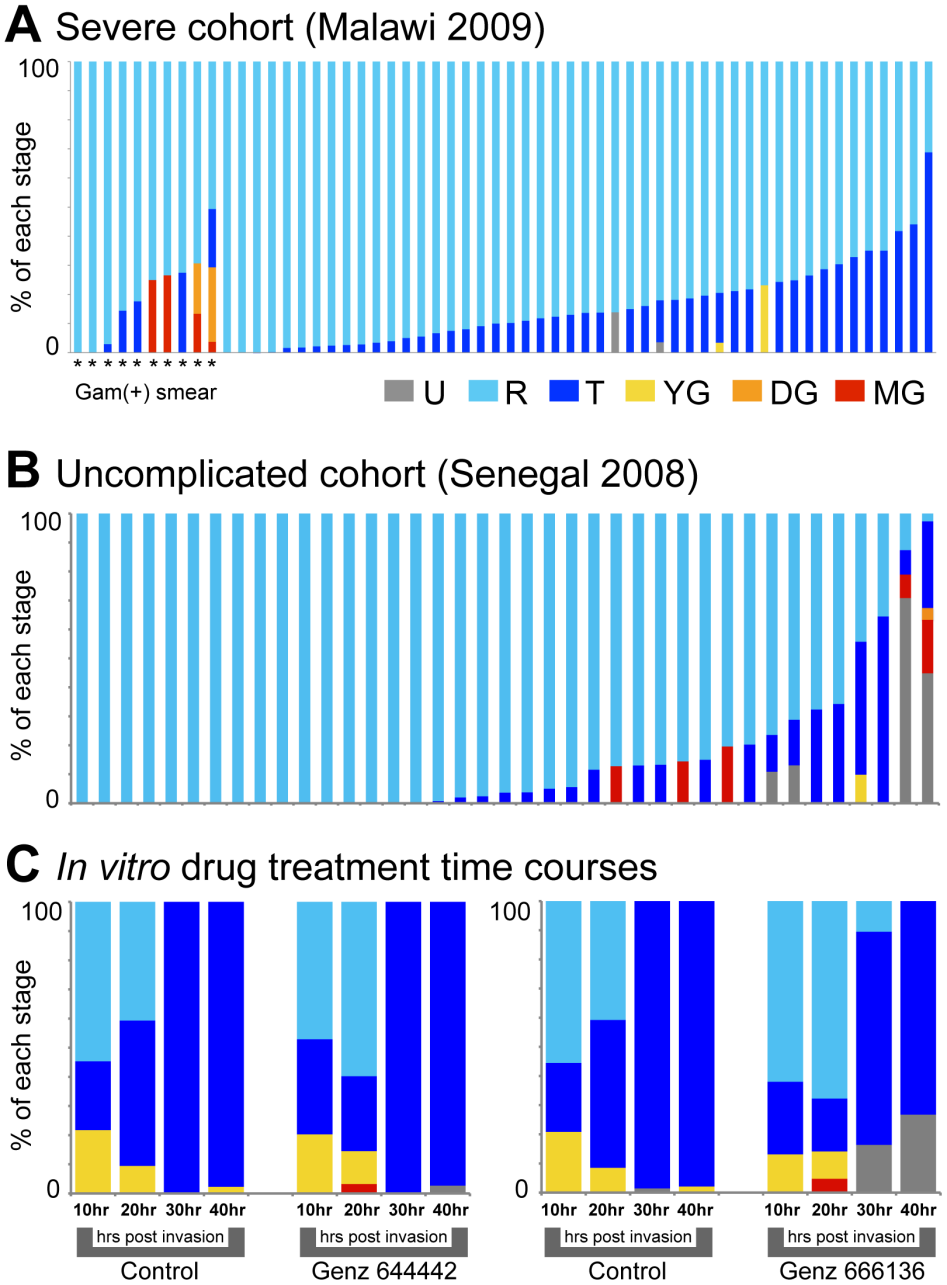
Application of microarray model to malaria patient cohorts and drug perturbation time courses. (A) Model inferences for 58 pediatric severe malaria patients from Blantyre, Malawi. Stars indicate subjects in which at least one gametocyte was observed by thick smear examination, a particularly sensitive assay (14 patients). (B) Model inferences for 39 adult uncomplicated patient samples from Dakar, Senegal. (C) Model inferences for *in vitro* time course experiments in which samples were taken at 10, 20, 30, and 40 hours post-invasion. Time courses were performed in the presence of one of two antimalarial compounds, Genz-666136 and Genz-644442, or under normal growth conditions (control).

With a set of six sentinel markers identified, we proceeded to test their ability to predict stage distributions *in silico*, first using cross-validation over the available labeled microarray data (Figure 1D-4). Having fit the complete regression model to establish β*_g,s_* parameters, we subsequently retained only those six markers' values for prediction of a sample's stage composition. This was done by solving for X_s_, the percent composition of each stage, while employing a quadratic programming approach to constrain the proportions of these stages to sum to 1. Making predictions based on gene expression from our selected marker set, we successfully predict the transition from asexual to sexual development across the life cycle time course (Figure 3 A/B). Prediction errors generated through cross-validation were minimal, with total per stage, per sample, root mean squared error of 0.195 (Figure 3C).

### Application of the model to *in vivo* malaria cohort and *in vitro* drug perturbation microarray samples

To gauge how this modeling process performed on patient microarray samples, we applied it to microarray data from two patient cohorts, i) a previously published cohort of severe malaria patients from Blantyre, Malawi collected in 2009 [23] and ii) a cohort of uncomplicated malaria patients from Thies, Senegal collected in 2008. While no staging information was available for the Senegal patients, a subset of Malawi patients were previously identified as gametocyte-positive by thick smears. The model inferred that the majority of patients from both cohorts have a strong ring-dominated profile, with the next largest subset being late asexual stages (trophozoites and schizonts) (Figure 4A/B). For the 10 Malawi samples in which gametocytes were observed by thick smear, our model correctly identifies 4 (40%) as such, with 0 false positive developing or mature gametocytes predicted among the 48 thick smear-negative patients (Figure 4A). Interestingly, two thick smear-negative patients are predicted to have young gametocytes, which are difficult to identify by thick smear microscopy due to their morphological similarities with asexual stages.

A subset of the uncomplicated malaria patients from Senegal were also predicted to be gametocyte carriers (6 of mature, 1 of developing and 1 of young gametocytes) (Figure 4B). As microscopy-based information was unavailable for the Senegalese cohort, we assessed how our gametocyte inferences correlated with patient parameters. Of the 6 parameters we measured for this cohort, illness duration and hematocrit differed significantly between the group of patients inferred to be gametocyte carriers and those inferred to be gametocyte-negative. The former had a longer duration of illness (6.33 days±1.02 SEM) than the latter (3.84 days±0.25 SEM, t-test p = 0.0014) as well as a lower hematocrit measured in percent cell volume (34.86%±2.17 SEM) than the latter (40.41%±1.06 SEM, t-test p = 0.031) (Supplementary Table S4). This finding agrees with published data on clinical correlates of gametocyte carriage: long illness duration (greater than 2 days) and anemia (hematocrit less than 30%) were both independently found to be risk factors of gametocytemia in uncomplicated malaria [24].

#### Evaluation of drug treatment on parasite stage distribution *in vitro*

We concluded validation of the microarray model by using it to profile the effects of drug treatments on parasite stage distribution. This application is of particular interest as it was recently demonstrated that currently used anti-malarial treatments including artemesinin combination therapy (ACT) have limited efficacy against mature gametocytes [25]. As a proof-of-concept we performed *in vitro* time course experiments for subsequent microarray analysis with a gametocyte-producing line of *P. falciparum* (3D7) in which parasites were grown in the presence of two experimental antimalarial compounds, Genz-666136 and Genz-644442. Genz-666136 is known to inhibit parasite dihydroorotate dehydrogenase (DHODH) enzyme, which catalyzes the rate-limiting step in *de novo* pyrimidine biosynthesis [26]. Genz-644442 was identified as a potent antimalarial compound with unknown target in a recent small molecule screen [27]. *Plasmodium* sexual differentiation in response to these compounds has not been previously explored.

In both the treated and untreated control samples, our model predicted an initial, subsequently decreasing fraction of the population to consist of young gametocytes (Figure 4C). The presence of young gametocytes is likely due to increased conversion of gametocytes in response to growth at the relatively high starting parasitemia used, an established induction factor of *in vitro* gametocyte development [28]. Mature gametocytes appeared in both drug-treated time course experiments 20 hours post invasion, while in the control experiment, we observed an expected progression from ring to trophozoite stages. The presence of mature gametocytes at 20 hours could reflect a differential killing effect of the drug on asexual stages versus mature gametocytes; this would induce exactly the inferred increase in relative proportion of gametocytes. A drug-induced stalling of asexual development is predicted in both time courses, as evidenced by the decreasing fraction of late asexual stages from 10 to 20 hours under drug treatment, compared with a constantly increasing late asexual fraction in controls (Figure 4C).

Treatment with both compounds, but to a greater extent with Genz-666136, resulted in an increasing proportion of transcriptional signature that could not be assigned to one of our stage categories (shown as “unknown”, gray in Figure 4C). It is possible that the increase in unknown transcriptional signature corresponds to an increase in dying parasites across the drug-treated growth experiment. By microscopy, stalled and dying asexual parasites could be observed in both drug-treated time courses.

### Application of the model for qRT-PCR–based inference of stage composition *in vitro* and in malaria patients

We next sought to test a variation of the microarray-based model for application to transcriptional measurements obtained by PCR, which might eventually be more appropriate for a field assay. As no MG marker that achieved our filtering criteria (see Figure 1C) for qRT-PCR also matched both the high expression levels and stage-specificity of the existing *Pfs25* marker for gametocyte detection, we assessed the utility of the YG and DG markers in combination for the prediction of immature and mature gametocyte quantities when applying the model to qRT-PCR data. Specifically, *PF14_0748* and *PF14_0367* were likely to represent immature (IG) and mature (MG) gametocytes in combination, as *PF14_0367* had a β*_g,s_* parameter similar to that of *Pfs25* in mature gametocyte stages. We therefore cross-validated this 5-marker PCR set (Table 1) comparably to the 6-marker microarray set, using the *in vitro* microarray time courses as described above. The simplified model remained able to predict stage distribution accurately, with a root mean squared error comparable to that of the 6-marker model (Supplementary Figure S1).

**Table 1:** Genes used in qRT-PCR assay.

Name	Accession	Stage-Specificity	PCR Efficiency	Limit of Detection
Skeleton-binding protein 1 (SBP1)	*PFE0065w*	ring	90.43%	10^1^ pg cDNA (appx 30 rings)
Alpha-beta hydrolase, putative	*PF10_0020*	trophozoite/schizont	87.82%	10^3^ pg cDNA (appx 1000 troph/schizonts)
Plasmodium exported protein (PHISTa)	*PF14_0748*	early - mid gametocyte	92.17%	10^2^ pg cDNA (appx 30 immature gametocytes)
Conserved Plasmodium protein	*PF14_0367*	mid - late gametocyte	89.77%	10^3^ pg cDNA (appx 20 mature gametocytes)
Conserved Plasmodium protein	*PF11_0209*	all stages	92.06%	10^2^ pg cDNA (appx 500 total parasites)

Details of the qRT-PCR compatible marker set selected by our combined filtering process. PCR efficiency was calculated based on the slope of the line after running a series of 10-fold dilutions of mixed-stage cDNA (Figure S2A). Limit of detection was calculated based on the number of parasite stages estimated to be present in the last dilution where the marker was detected. Detection limit ranged from 10^1^–10^3^ pg cDNA, corresponding to approximately 20–200 cells, depending on the stage.

In order to create a qRT-PCR assay for our sentinel transcripts, we designed exon-exon junction spanning primers (distinguishing transcripts from genomic DNA) and sequence-specific probes (distinguishing transcripts from non-specific background amplification). Following confirmation that our primer/probe sets selectively amplified cDNA and not genomic DNA or non-specific products, we validated the stage-specific expression using *in vitro*-derived asexual and sexual stage RNA (Table 1, and supplementary Figure S2A, and Table S5 for optimization and validation of qRT-PCR parameters). For these experiments, we used the gametocyte-producing reference line 3D7 and a gametocyte-deficient clone thereof (termed F12 [29]) to confirm the stage-specificity of each of our sentinel markers. Normalized expression data from time courses of 3D7 and F12 confirmed stage-specificity of our sentinel marker set (Supplementary Figure S2B). The asexual markers alternate with respect to time points in which there were predominately rings or trophozoites and schizonts in the culture, with similar results for both the F12 and 3D7 lines. The sexual markers demonstrate stage-specificity within the 3D7 time course and no appreciable expression in the F12 line once normalized. Specifically, *PF14_0748* expression is detected in the early and mid gametocyte time points, while *PF14_0367* expression is detected in both mid and late time points.

#### *In vitro* stage prediction using qRT-PCR

**Figure 5:**
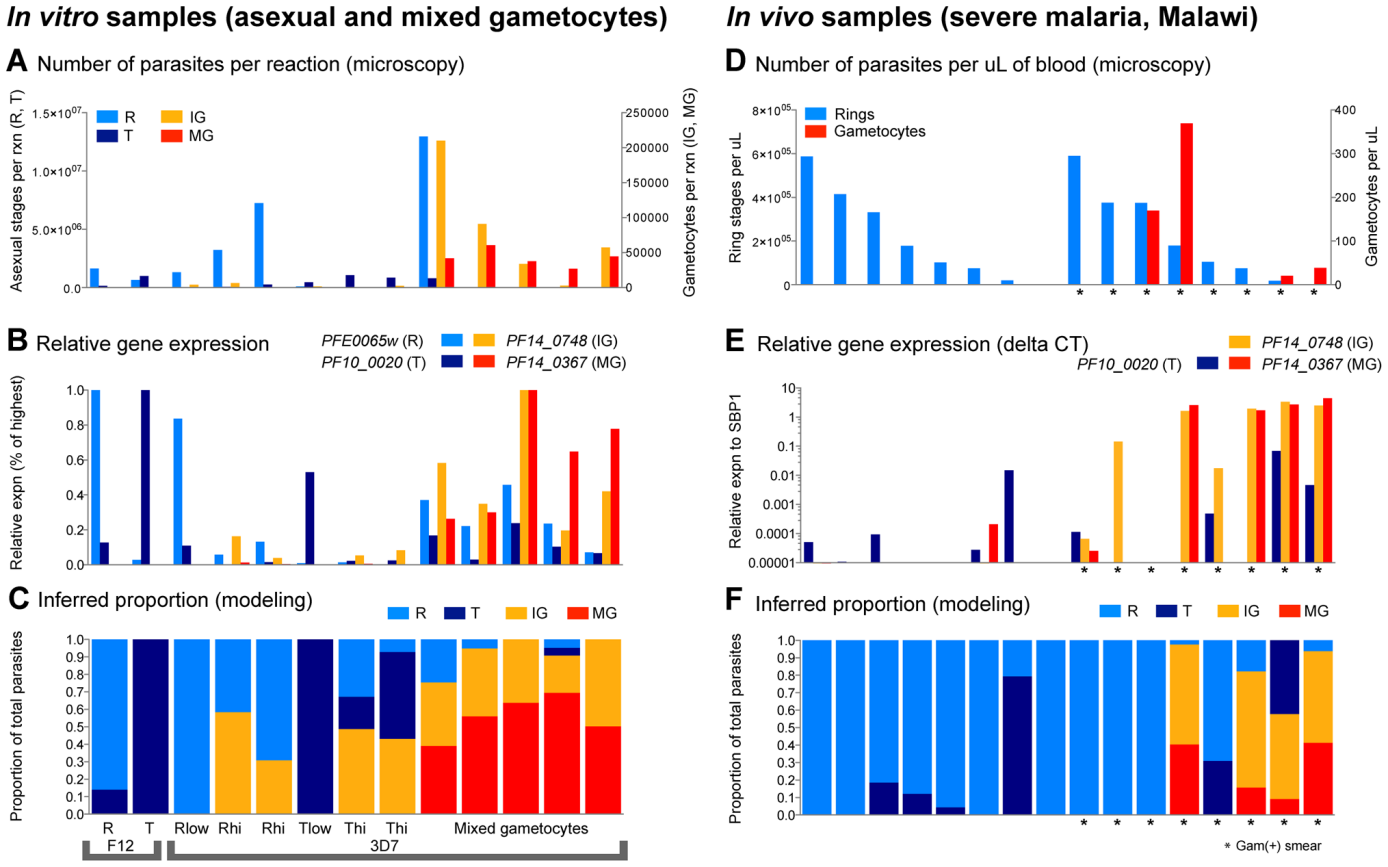
qRT-PCR assay optimization. (A) We collected and analyzed a range of *in vitro* time points with varying contributions of asexual and sexual stages, from both gametocyte-producing and non-producing lines of 3D7. Absolute number of parasites stages that went into each qRT-PCR reaction well is plotted. (B) Relative qRT-PCR-based gene expression of stage-specific markers for R, T, IG and MG are shown for time points corresponding vertically to those in part A. (C) Inferred proportion of each stage in the total parasite load (model predictions) are shown corresponding vertically to the time points in A and B, plotted as a percentage of total parasites in that sample. (D) *In vivo* peripheral blood samples from severe malaria patients in Blantyre, Malawi were collected and analyzed. Absolute numbers of parasites stages per µL of blood, as determined by microscopy, are plotted. (E) Relative qRT-PCR-based gene expression of stage-specific markers for T, IG and MG (normalized to *SBP1*) is shown for time points corresponding vertically to those in part D. (F) Inferred proportion of each stage (model predictions) are shown corresponding vertically to the time points in D and E. Stars indicate subjects in which gametocytes were observed by highly sensitive thick smear examination (one or more gametocytes in 100 high power fields).

Having validated the stage-specificity of each marker *in vitro* and tested their predictive-ability as a set *in silico*, we next compiled a larger set of *in vitro* samples with known distributions of parasite life cycle stages in order to tune a set of model parameters for stage prediction specifically using qRT-PCR-based expression measurements. For this purpose, we used the data from our *in vitro* validation on the 3D7 and F12 lines, in combination with additional *in vitro* samples generated from across a 48-hour period of asexual development, and a two-week period of gametocyte conversion. For the additional data points, we used a transgenic line in 3D7 background, termed 164/GFP, that expresses fluorescent protein under the gametocyte-specific *PF10_0164* promoter [22]. This parasite line enabled us to determine stage composition with high accuracy by both Giemsa stain and fluorescence microscopy throughout gametocyte development and starting at the earliest stages (Figure 5 A/B). The model was trained on these qRT-PCR datasets as described earlier for microarray data, again determining the contribution *β_g,s_* of each stage to our five markers' expression and performing cross-validation to evaluate the final prediction error rates per stage. Again, we see that the model accurately predicts the absence of sexual stages in F12 and low parasitemia cultures of 3D7 while predicting young gametocytes in the high parasitemia 3D7 cultures (Figure 5C), similar to the distribution seen in the early time points of the drug profiling experiments (see Figure 4C). Developing and mature gametocytes are observed in the later mixed gametocyte induction cultures (Figure 5C).

#### Application of the qRT-PCR and model predictions to a cohort of severe malaria cases

To finally test the sensitivity of our system in an epidemiological context, peripheral blood samples from a cohort of severe malaria patients in Blantyre, Malawi were collected over the course of the malaria transmission season in 2011 (see Table 2 for patient cohort characteristics). Smears were quantified using standard methods of ring quantification (thin smear microscopy and grid) and detailed gametocyte quantification (highly sensitive thick smear microscopy screen of 100 high power fields for positivity, then quantification against 500 white blood cells, see Figure 5D).

**Table 2:** Admission characteristics of severe malaria patients tested by qRT-PCR, Blantyre, Malawi, 2011.

**Demographics**	
Mean age (months)	52.9
Age range (months)	5–156
Gender	47.3% female
**Parasitemia**	
Geometric mean parasite density/µl [95% CI]	48349 [29051–80466]
Median parasite density/µl	74800
Parasite density range/µl	69–945500
**Gametocytemia**	
Gametocyte prevalence (Screen of 100 HPF on thick smear)	11.9% (8/67)
Gametocyte density range/µl (Counts per 500 WBC on thick smear)	0–370

Blood was sampled from participants who met the clinical case description of cerebral malaria during the malaria transmission season in 2011. All patients were from Blantyre, Malawi and surrounding areas. Parasitemia was measured by microscopy and qRT-PCR was performed on an RNA sample stored in trizol.

Of the 86 samples examined, 8 were gametocyte-positive by thick smear. We performed qRT-PCR on these samples and 8 matched gametocyte-negative samples with equivalent parasitemias. Seven of the 8 (88%) microscopy-positive gametocyte carriers were qRT-PCR positive for one or both gametocyte markers, while only three of the 8 (38%) matched microscopy-negative individuals were positive for either gametocyte marker, with highest levels of expression observed in the microscopy-positive gametocyte carriers (Figures 5E). This provides an approximate baseline of error rates for existing single-marker qRT-PCR approaches.

In comparison, our model provided additional detail, first predicting that most patients have a ring-dominated profile as expected in peripheral blood (Figure 5F). A number of individuals were predicted to have trophozoites and schizonts, which has been shown to be associated with severe malaria [30]. The model predicted gametocyte fractions in four patients, all of which were gametocyte-positive by thick smear. We thus achieve a comparable false negative rate (50%) and a higher true positive rate (100%) to single-marker qPCR as compared to standard thick-smear microscopy.

## Discussion

Several highly sensitive single-marker molecular assays are currently used to detect *Plasmodium* gametocytes. None of these existing tools have been appropriate for detection and quantification of the relevant range of parasite stages present during infection, however, due primarily to the lack of a sufficiently broad panel of stage-specific markers. Further, since malaria parasite populations exist as mixtures of the different phases of the life cycle, assays combining multiple markers require customized computational analysis methods for dealing with this complexity. We combined the development of such a bioinformatic deconvolution approach with panels of stage-specific, intron-containing markers appropriate both for microarray analysis and a newly developed qRT-PCR assay. This multi-marker platform enabled us not only to detect gametocyte carriers but primarily to infer the relative amounts of sexual and asexual stages within a sample. We provide an implementation of this platform for further development and application, particularly for refinement in field settings. This process can also be adapted bioinformatically by the exclusion or inclusion of markers to answer specific questions, such as determination of parasite sex ratios that are known to influence mosquito infectiousness [31].

Our deconvolution model provided the opportunity to define stage-specific gene sets and to characterize the biology of these stages' expression programs using tools such as GSEA, even in the absence of transcriptional data from pure stage populations. For example, our GSEA analysis confirms earlier studies that suggested increased mitochondrial and lipid metabolism during gametocyte development [3], [32]. Interestingly, the analysis also suggests significant enrichment of several markers related to endocytic trafficking in late gametocyte development but not in any other parasite stage. The biological significance of this observation remains to be determined. To put such findings into context and ultimately describe the gametocyte transcriptome at high resolution, a systematic transcriptional re-analysis of the entire *P. falciparum* gametocyte cycle using isolated and synchronous gametocyte stages will be required.

Transcriptional approaches have significantly increased the sensitivity of gametocyte detection in field-compatible assays [8], [9], [33], [34], [35]. However, these have been limited to either (i) qualitative assessments of multiple gametocyte markers, i.e. RT-PCR of immature and mature gametocyte markers [35], or (ii) quantitative assessments of mature gametocytes only, i.e. QT-NASBA of the gamete surface antigen *Pfs25* [8]. In order to properly define the reservoir of parasite and gametocyte carriers in the field, it is imperative to determine both the absolute parasite burden and the stage composition of parasites in the blood circulation. Challenges have prevented the development of a diagnostic that can measure the latter, such as (i) the lack of transcriptional analysis methods to identify gametocytes with high specificity in a sample containing a mixture of stages, (ii) the lack of validated immature gametocyte markers, and (iii) the lack of known intron-containing qRT-PCR compatible markers for all stages. We tackled these challenges by developing a model specific to the quantification process and ensuring that it was compatible with both microarray and qRT-PCR measurements. This is distinct, of course, from models that would focus only on sensitivity and specificity of gametocyte *detection* from such data, which represent a potentially fruitful course of future computational investigation. Instead, by incorporating relative expression values of the markers, the model allowed us both to identify a subset of patients as gametocyte carriers and to additionally quantify sub-categories of immature and mature gametocyte fractions within the mixture of stages in the bloodstream.

Following validation of our model on samples for which stage composition was known, we applied our model to two microarray data sets in which stage composition was unknown: (i) a cohort of uncomplicated malaria patients, and (ii) two *in vitro* growth experiments in the presence of drug. In the former, we found that both mean illness duration and hematocrit differed between inferred gametocyte carriers and non-carriers, in agreement with published data demonstrating that long illness duration and low hematocrit is linked to gametocyte carriage [24]. In the latter, we observed an increase in the fraction of mature gametocytes as well as unexplained transcriptional signature upon the addition of drug treatment to parasites. The enrichment of mature rather than young gametocytes in response to drug treatment suggests that the drug selectively kills asexual stages, leaving gametocytes unaffected rather than inducing the development of new young gametocytes. The increase in unexplained signatures likely indicates the transition to unhealthy, dying parasite fractions. These applications demonstrate the range of potential uses for this inference tool.

As the exon-exon junction spanning primer/probe sets for 5 markers designed here represent the first attempt at a multi-marker gametocyte-staged qRT-PCR assay, further modeling of PCR-specific measurement error and careful standardization of experimental protocol for this difficult task will both improve field inferences. Like the microarray expression model, however, this model successfully recapitulated the transition from asexual to sexual development across multiple *in vitro* experiments even on first application. When used initially *in vivo* for blood samples from a cohort of children with severe malaria in Malawi, the system successfully identified a subset of patients as immature and/or mature gametocyte carriers. Because immature gametocytes in particular are present in the body several days before the more mature forms emerge, our approach for detecting them could be used in further investigations into factors that influence gametocyte conversion *in vivo*.

The assay and algorithm framework presented here has potential for use in epidemiological studies such as those of asymptomatic carriers, who likely represent a major reservoir for malaria transmission. Multiple such studies are already ongoing and will yield additional samples to further optimize computational models of gametocyte differentiation. This is also true of data generated from other sensitive expression platforms such as glass-slide arrays or Nanostring. The inference process may thus have applications in better understanding the natural progression of malaria in the human host, by identifying gametocytes earlier in the course of infection and determining the impact of specific drug treatments on gametocyte development. By scaling to future population-level screens, the resulting information will help better characterize the epidemiology of gametocytemia based on malaria transmission intensity, geography, climate and season.

## Materials and Methods

### Ethics statement

The institutional review boards of the Harvard School of Public Health, Brigham and Women's Hospital, the University of Malawi College of Medicine, and the Ministry of Health in Senegal approved all or parts of this study. Consent was obtained from the patient or a child's guardian.

### Patients and sample collection

#### Malawi patient isolates

Patients who enrolled in an ongoing severe malaria study [36] at the Queen Elizabeth Central Hospital during the 2009 and 2011 transmission season were included in this study. These patients were between the ages of 1 month to 14 years of age and came from Blantyre, Malawi and surrounding areas, where transmission is high and seasonal. All patients enrolled in the study met the clinical criteria for severe malaria, and severity was classified by Blantyre Coma Score [37]. The majority of patients were treated with an antimalarial drug (majority received quinine) within the 24 hours prior to admission. Parent or guardians of all children enrolled in the study were consented in writing in their own language by local native-speaking healthcare staff (nurse or doctor). The samples from the 2009 cohort have been described in detail in a recent publication [23]. For the 2011 cohort, a venous blood sample was drawn at admission and a 500 µl sample of whole blood was added directly to Tri-Reagent BD (Molecular Research Center), mixed vigorously and stored at −80 C until processing. Simultaneously, thick and thin smears were collected and stored for later processing. Patients were classified as “gametocyte-positive” if they had 1 or more gametocytes in 100 thick smear high power fields (HPF). For standard gametocyte quantification by thick smear, we quantified gametocytes per 500 white blood cells (WBC).

#### Senegal patient isolates

Patients who enrolled in a study of uncomplicated malaria in Thies, a low malaria endemicity suburb of Dakar, Senegal in October 2008 during transmission season were included in this study. Patients who presented to Section de Lutte Antiparasitaire de Thies, with signs and symptoms of malaria were offered enrollment if they had microscopic confirmation of malaria infection and had mild symptoms. At the time of admission, a blood sample was taken from which 4.5 mL was transferred into Tri-Reagent BD (Molecular Research Center), shaken vigorously for 15 seconds, and frozen at −80°C for transcriptional analyses. General demographic data, history of illness and hematocrit were recorded. Samples were transported to HSPH in a liquid nitrogen dry shipper, thawed in a room temperature water bath and RNA isolated according to manufacturer's instructions (Molecular Research Center). Simultaneously, thin smears were collected and stored for later processing.

### *P. falciparum* asexual and sexual *in vitro* culture

A transgenic line, 164/GFP, of a gametocyte-producing clone of the 3D7 strain of *P. falciparum* was used to produce the mixed stage samples for model training and validation. This transgenic line, which aided in the quantification of gametocyte stages, produces stage-specific GFP expression under the *PF10_0164* gene promoter, as described previously [22]. A previously characterized non gametocyte-producing clone, F12, of the 3D7 strain was used to confirm stage-specificity of gametocyte markers [29]. Culture conditions were as described previously [38], maintaining the parasite line in O+ blood at 4% hematocrit in RPMI-1640 media supplemented with 10% human serum. Cultures were kept at 37°C in a chamber containing mixed gas (5% CO_2_, 5% O_2_, 90% N_2_). Prior to induction, asexual parasite cultures were synchronized for two cycles with 5% D-sorbitol [39], and subsequently induction of gametocytogenesis was performed according to the Fivelman protocol [28]. Briefly, asexual parasites were grown to a high parasitemia in the presence of partially spent (“conditioned”) medium, and then sub-cultured at the schizont stage into new dishes containing fresh media and erythrocytes. One of two methods was used to reduce the amount of asexual stages in the cultures: Treatment with D-sorbitol was applied on two days later to lyse asexual trophozoite/schizont stages and selectively enrich for unaffected early gametocytes, or N-Acetyl glucosamine was added to the medium one day later and every subsequent day to selectively kill asexual stages.

### *P. falciparum* drug perturbations

A 3D7 line was used to study the effect of drug perturbations on parasite growth. Culture conditions were performed as described above. Asexual parasite cultures were synchronized for three cycles with 5% D-sorbitol, and expanded to a parasitemia of 5–6%. Hematocrit was increased from 3 to 6% at the late schizont stage using fresh blood. Upon reinvasion drugs were added to the culture at a concentration of 5×IC_50_. Drug-treated and control parasites were harvested at 10, 20, 30, and 40 hours post-invasion and RNA was extracted (Qiagen).

### Standard and fluorescence microscopy

In order to accurately quantify the stage distribution of parasites in our *in vitro* samples, we used a combination of standard and fluorescence microscopy. Parasite stage distribution was monitored throughout the parasite synchronization and induction protocol using Wright's Giemsa stain applied to thin blood smears. Quantification of asexual rings and trophozoite stages, as well as developing and mature sexual stages was done directly by light microscopy. In order to quantify early stages of sexual development that are morphologically similar to asexual stages, we used a combination of live imaging and immunofluorescence microscopy. Live imaging was performed using the transgenic 164/GFP line. Parasites were analyzed using the FITC channel on an inverted epifluorescence microscope (Zeiss) and quantification was done of the proportion of GFP(+) parasites out of the total number of Hoechst (+) parasites. Immunofluorescence assays were performed with cell monolayers on glass slides, prepared as described previously [40]. For labeling with the constitutive gametocyte marker Pfs16, slides were fixed in ice-cold methanol, blocked with 5% nonfat dry milk powder, incubated with polyclonal mouse antibody against Pfs16 (1∶2500) [41], washed and incubated with a secondary antibody conjugated to Alexa 488. Parasite nuclei were labeled with DAPI and quantification was done on the proportion of FITC(+) parasites out of the total number of DAPI(+) parasites. For time points in which we had data from both live and immunofluorescence experiments [41], the quantification of early gametocytes from both methods was averaged to give the final amount.

### Quantitative reverse-transcriptase PCR assay

#### Primer & probe design

We have developed a quantitative reverse-transcriptase PCR (qRT-PCR) assay for the quantification of five key gene transcripts. The assay includes primers and probes designed against the markers described in the Results section. Primers and probes were designed by hand using the PrimerExpress software (Applied Biosystems) and following recommended guidelines for qRT-PCR primer and probe design. Primers were specifically designed to cross exon-exon junctions, so as to reduce genomic DNA amplification. In addition both primers and probes were checked for homology against *Plasmodium* or human homologous sequences using PlasmoDB and NCBI Blast in order to eliminate the chances of non-specific amplification (see also Supplementary Table S5 for primer and probe validation).

#### RNA extraction, DNAse digest and reverse transcription

RNA from mixed stage cultures was preserved and extracted and processed as previously described [42]. Briefly, samples were stored in TriReagent (Molecular Research Center) until use. For sample processing, RNA was extracted by the phenol-chloroform method followed by DNAse digest (Ambion), and a second phenol-chloroform extraction for protein removal and sample concentration. For first strand synthesis we used the SuperScript III First Strand Synthesis kit (Invitrogen).

#### qRT-PCR assay optimization

Amplification of the correct target sequence was confirmed by gel electrophoresis and melt curve analysis using SYBR Green (BioRad). The ability of primer pairs to discriminately amplify the cDNA product was determined by performing qRT-PCR on mixed stage *P. falciparum* cDNA and genomic DNA (same extraction, minus DNAse digest and reverse transcription for genomic DNA). The possibility of non-specific amplification with host template was ruled out by performing qRT-PCR on cDNA and genomic DNA from a human whole blood sample from which GAPDH was successfully amplified (data not shown). Primer pair efficiencies were determined by calculating the slope of the crossing threshold (CT) values on 10-fold serial dilutions of mixed stage cDNA (Supplementary Table S6).

#### Sensitivity assessment

Sensitivity of this assay was determined using 10-fold serial dilutions of mixed stage cDNA. Sensitivity was estimated by calculating the amount of parasites of each stage that were present in the sample at the limit of detection by qRT-PCR.

### Microarray expression analysis

RNA (from peripheral blood of Senegalese patients and cultured *in vitro* drug perturbations) was assessed by Bioanalyzer (Agilent), and high quality RNA samples were labeled and hybridized to an oligonucleotide array (Affymetrix) custom-designed for the *P. falciparum* 3D7 genome, PlasmoFB, as published previously [5]. The raw CEL files were condensed into GCT expression files using RMA and the default parameter settings in ExpressionFileCreator in GenePattern [43].

### Development and validation of the constrained linear regression model

#### Development

A constrained linear regression model was constructed to estimate the contribution of each stage to the overall transcript abundance from training data

The total transcript abundance *y_g_* for each gene *g* was modeled as a mixture of its abundance in each specific stage U,R,T,IG or YG+DG and MG. The mixture fraction *x_s_* represents the proportion of parasites in each stage *s* and is thus constrained between zero and one.

#### Microarray model training to establish genome-wide stage-specific expression

This model was first fit to estimate the contribution of each stage to each transcript's abundance, parameters *β_g,s_*. Labeled training data were obtained from a published *in vitro* time course [3], [5] in which the stage-specific parasite fractions *x_s_* were known from fluorescence microscopy. The model was fit to these training data using lm, the linear model method without an intercept, in R, resulting in a table of 5,159 genes across the 5 erythrocyte stage-specific parameters (Supplementary Table S1).

#### Gene set enrichment analysis

GSEA was performed using the pre-ranked *β_g,s_* parameters determined in this manner in combination with a newly derived set of *P. falciparum*-specific gene sets. For this purpose *P. falciparum* Gene Ontology [13] leaf annotations were obtained from PlasmoDB and propagated into the ontology using the Sleipnir functional genomics library [44]. Gene sets containing <2 genes were removed, and overlapping gene sets were merged (combining the top 10% of gene set pairs ranked by the fraction of shared genes relative to the total size of the smaller set). These gene sets were finally combined with the *P. falciparum* pathways from KEGG [45]. Enrichment of the resulting pathway sets (Supplementary Table S3) was then assessed in the five pre-ranked parameter lists using GSEA over 1000 bootstraps.

### Model application to predict stage distribution

Given the learned model parameters *β_g,s_* from stage-labeled data with known *x_s_*, the model was inverted to infer the unknown stage distributions *x_s_* in new samples. A quadratic programming approach was used to solve the system of linear equations with the constraint that the proportions of all stages must sum to 1 and that each stage contributes a non-negative fraction of expression:
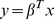


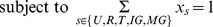


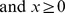



We implemented this process in the R function quadprog and solved for the stage distributions using the sets of six (for microarrays) or five (for PCR data) markers ultimately selected as follows.

#### Co-normalization of reference and inference data sets

*Microarray data sets.* For model inference in microarray data, missing values remaining in input microarrays were imputed per dataset using the row mean across all samples. For each set of two or more training and inference datasets obtained in different batches, values used in the modeling process were co-normalized using quantile normalization. Datasets were first merged, retaining only gene names common among both datasets and the resulting merged data was jointly quantile normalized.

*qRT-PCR data sets.* Similarly for qRT-PCR expression values, raw values were delogged using the formula to bring the data into a range of log-scaled abundance counts comparable to microarray samples. Missing values were imputed to the lowest detectable limit on our qRT-PCR assay, which corresponds to a CT value of 50 or 3.23 in delogged expression space. Again considering sets of reference and inference data together, data were median normalized, such that all samples had the same median value (i.e. adjusting expression values by subtracting the column median and adding the global median).

#### Stepwise backward marker selection

To identify reduced marker sets appropriate for inference, we used the model's inference process to perform *in silico* marker selection based on greatest accuracy of stage inference in our labeled microarray training data. Iteratively, model inference was performed on the total labeled microarray time courses using the complete transcriptome. Each individual gene was removed, the model re-applied, and the marker inducing the smallest increase in inference error removed. These steps were repeated to a minimum of five markers, and the entire process repeated 15000 times. The resulting whole transcriptome rankings were averaged to assign a rank-average selection preference to retention of each *Plasmodium* gene as one criterion for our reduced marker set.

#### Bootstrap cross validation

The model's accuracy in each labeled microarray and qPCR training set was evaluated by 10-fold bootstrap cross validation. For each fold, one third of conditions were selected for holdout with replacement to form a test set. The model was fit as above to the remaining training data and its accuracy in predicting new expression samples was calculated using a per stage root mean square error in the test set.

## Supporting Information

Figure S1 **Performance of the 5 marker model on published microarray data sets.** (A). Actual and (B) inferred stage distributions across five microarray time courses (two asexual and three sexual) with reference stage distributions determined by microscopy. Five markers were used to make these predictions (Table 1). (C). Bootstrap cross-validation of error rates expected per-stage in model inferences. Violin plots show expected density, with internal boxplots detailing the 25^th^–75^th^ percentiles and 1.5× fences.
(TIF)

Figure S2 **qRT-PCR assay optimization.** (A) Efficiency of qRT-PCR reactions using 10-fold dilutions of mixed parasite cDNA. 4 to 6 dilutions were assessed for each primer, and efficiencies were in the acceptable range for all 5 primers (87–92%). R^2^-values were all greater than 0.96. Technical variation between replicates was very low: the average standard deviation between technical replicates was 0.243 and ranged between 0.01 and 1.543. (B) Stage-specificity of qRT-PCR markers. Using two clones of 3D7, F12 (gametocyte deficient) and wild type (gametocyte producer), we performed *in vitro* gametocyte inductions and collected parasite samples for microscopy and qRT-PCR at days −1, 0, 1, 5, 10 according to the Fivelman et al protocol [28]. Results, displayed as relative expression normalized to constitutively expressed marker *PF11_0209*, confirm stage-specificity of markers.
(TIF)

Table S1 **Annotated gene list and metadata.** Annotations of the 5160 genes in the *P. falciparum* transcriptome used in the analysis, including the frequency of selection in our subsampling and backward selection steps, presence of an intron, contribution of expression to stage, determination of stage specificity, product description and population genetic parameters of total SNP counts, diversity and divergence.
(XLSX)

Table S2 **Complete GSEA results per stage.** Results for each stage in our microarray model, wherein the per gene z-scored contributions of expression to that stage were ranked and were characterized for enrichment in functional pathways.
(XLSX)

Table S3 **GSEA gene sets.** Gold Standard Catalog of GO and Kegg pathways obtained from individual GO slims from PlasmoDB and the GO ontology integrated into the GO hierarchical structure.
(XLSX)

Table S4 **Clinical parameter data for Senegal cohort.** GraphPad Prism Version 6.0 was used to compare two groups (those inferred to have gametocytes and those not inferred to have gametocytes) for six continuous variables measured at admission: age, hematocrit, temperature, illness duration, height, and weight. A multiple t-test analysis was performed, analyzing each variable individually, and then using false discovery rate (Q = 0.25) to determine significance.
(DOCX)

Table S5 **Additional qRT-PCR assay optimization data.** Primers were specifically designed to cross exon-exon junctions, so as to reduce genomic DNA amplification, and were checked for homology against *Plasmodium* or human homologous sequences using PlasmoDB and NCBI Blast in order to eliminate the chances of non-specific amplification. Using our primer set with sequence-specific probes showed no cross-reactivity with genomic DNA or human templates. Our primer sets also greatly reduced the amount of genomic DNA amplification even using SYBR (CT>39 as compared with DNA-amplifying control marker at CT = 25), yet it was not zero.
(DOCX)

Table S6 **Primer and probe sequences used in qRT-PCR.** Sequences for the reverse and forward primers and minor groove-binding fluorescent probes used in the qRT-PCR assay.
(DOCX)
