# Supplementary figures and images for: Inferring Developmental Stage Composition from Gene Expression in Human Malaria
Source: PLoS Comput Biol. 2013 Dec 12;9(12):e1003392. doi: 10.1371/journal.pcbi.1003392 (PMC3861035; doi:10.1371/journal.pcbi.1003392)

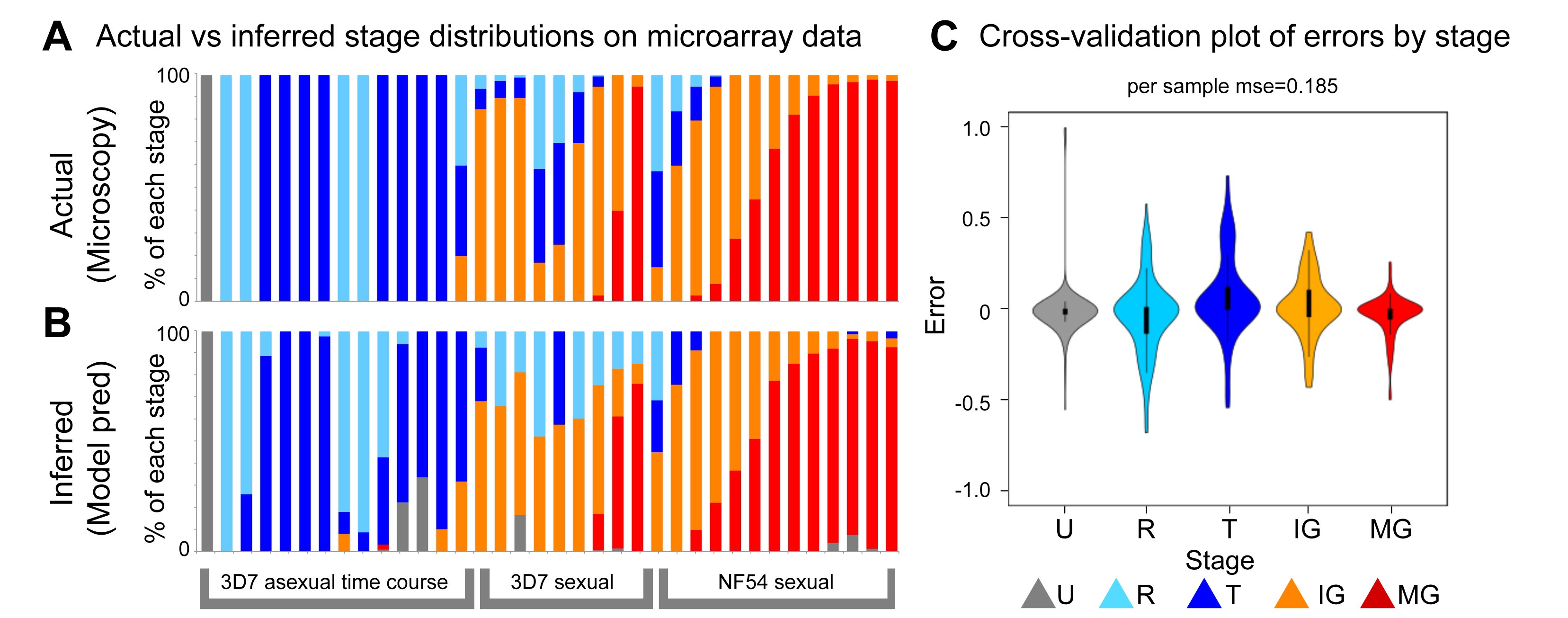

Supplement: Figure S1 — Performance of the 5 marker model on published microarray data sets. (A). Actual and (B) inferred stage distributions across five microarray time courses (two asexual and three sexual) with reference stage distributions determined by microscopy. Five markers were used to make these predictions (Table 1). (C). Bootstrap cross-validation of error rates expected per-stage in model inferences. Violin plots show expected density, with internal boxplots detailing the 25th–75th percentiles and 1.5× fences. (TIF) [file pcbi.1003392.s001.tif]

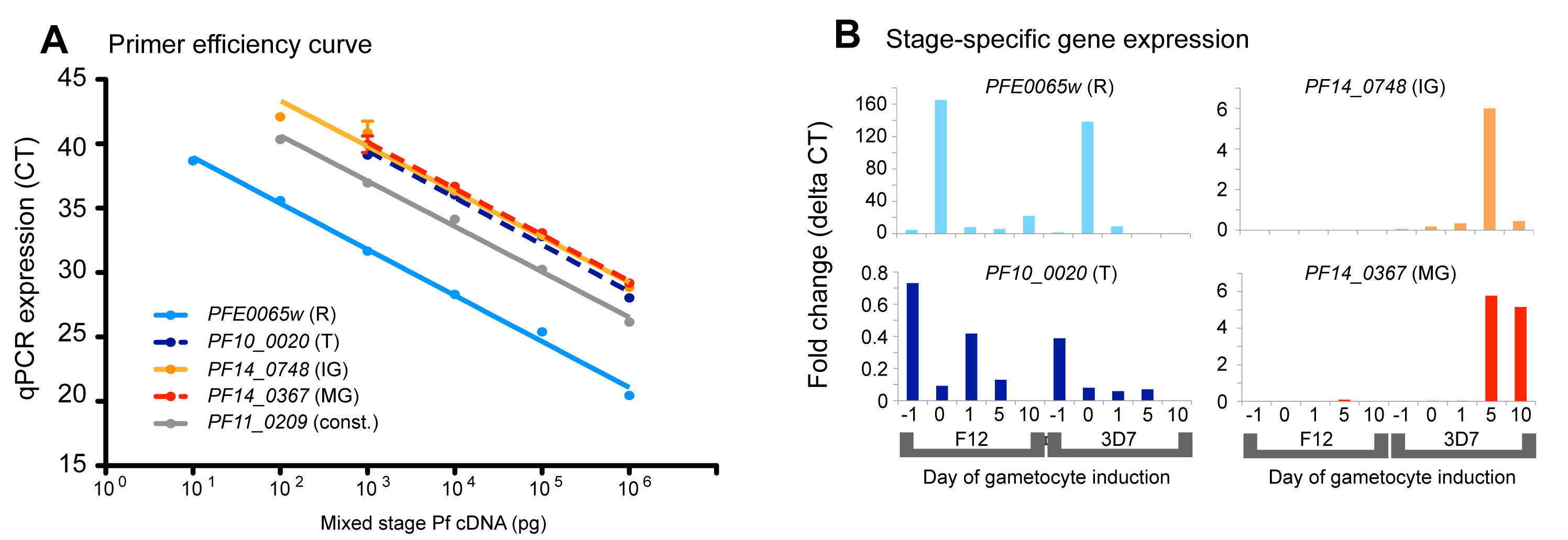

Supplement: Figure S2 — qRT-PCR assay optimization. (A) Efficiency of qRT-PCR reactions using 10-fold dilutions of mixed parasite cDNA. 4 to 6 dilutions were assessed for each primer, and efficiencies were in the acceptable range for all 5 primers (87–92%). R2-values were all greater than 0.96. Technical variation between replicates was very low: the average standard deviation between technical replicates was 0.243 and ranged between 0.01 and 1.543. (B) Stage-specificity of qRT-PCR markers. Using two clones of 3D7, F12 (gametocyte deficient) and wild type (gametocyte producer), we performed in vitro gametocyte inductions and collected parasite samples for microscopy and qRT-PCR at days −1, 0, 1, 5, 10 according to the Fivelman et al protocol [28]. Results, displayed as relative expression normalized to constitutively expressed marker PF11_0209, confirm stage-specificity of markers. (TIF) [file pcbi.1003392.s002.tif]
