# Supplementary material for: Inferring Developmental Stage Composition from Gene Expression in Human Malaria
Source: PLoS Comput Biol. 2013 Dec 12;9(12):e1003392. doi: 10.1371/journal.pcbi.1003392 (PMC3861035; doi:10.1371/journal.pcbi.1003392)
Supplement: Table S4 — Clinical parameter data for Senegal cohort. GraphPad Prism Version 6.0 was used to compare two groups (those inferred to have gametocytes and those not inferred to have gametocytes) for six continuous variables measured at admission: age, hematocrit, temperature, illness duration, height, and weight. A multiple t-test analysis was performed, analyzing each variable individually, and then using false discovery rate (Q = 0.25) to determine significance. (DOCX) [file pcbi.1003392.s006.docx]

|  | t-test | | Gametocyte (-) | | | Gametocyte (+) | | |
| --- | --- | --- | --- | --- | --- | --- | --- | --- |
|  | Significant  q = 0.25 | P value | Mean | SEM | N | Mean | SEM | N |
| Age (years) |  | 0.542917 | 23.6875 | 2.029737 | 32 | 26.85714 | 6.123446 | 7 |
| Hematocrit  (PCV) | * | 0.0310864 | 40.40625 | 1.055763 | 32 | 34.85714 | 2.164965 | 7 |
| Fever  (C) |  | 0.234388 | 38.88125 | 0.4496737 | 32 | 40.14286 | 0.8574207 | 7 |
| Illness duration (days) | * | 0.00144451 | 3.83871 | 0.2544096 | 31 | 6.333333 | 1.021981 | 6 |
| Height  (m) |  | 0.434628 | 1.683333 | 0.04033357 | 18 | 1.741667 | 0.03370626 | 6 |
| Weight (kg) |  | 0.748239 | 56.34615 | 3.347993 | 26 | 58.66667 | 2.703907 | 6 |
