# Supplementary material for: Inferring Developmental Stage Composition from Gene Expression in Human Malaria
Source: PLoS Comput Biol. 2013 Dec 12;9(12):e1003392. doi: 10.1371/journal.pcbi.1003392 (PMC3861035; doi:10.1371/journal.pcbi.1003392)
Supplement: Table S5 — Additional qRT-PCR assay optimization data. Primers were specifically designed to cross exon-exon junctions, so as to reduce genomic DNA amplification, and were checked for homology against Plasmodium or human homologous sequences using PlasmoDB and NCBI Blast in order to eliminate the chances of non-specific amplification. Using our primer set with sequence-specific probes showed no cross-reactivity with genomic DNA or human templates. Our primer sets also greatly reduced the amount of genomic DNA amplification even using SYBR (CT>39 as compared with DNA-amplifying control marker at CT = 25), yet it was not zero. (DOCX) [file pcbi.1003392.s007.docx]

| **Gene** | **Stage-Specificity** | **Pf cDNA**  **(mixed stage)** | | **Pf DNA** | | **Human cDNA** | | **Human DNA** | | **H2O** | |
| --- | --- | --- | --- | --- | --- | --- | --- | --- | --- | --- | --- |
| All reactions run to 45 cycles. | | Probe | SYBR | Probe | SYBR | Probe | SYBR | Probe | SYBR | Probe | SYBR |
| ***PFE0065w*** | ring | 20.17 | 15.85 | - | 39.71 | - | - | - | 42.89 | - | - |
| ***PF10_0020*** | trophozoite/  schizont | 30.50 | 25.82 | - | 44.64 | - | - | - | - | - | - |
| ***PF14_0748*** | early - mid gametocyte | 32.29 | 27.04 | - | 40.02 | - | - | - | - | - | - |
| ***PF14_0367*** | mid - late gametocyte | 33.31 | 29.08 | - | 40.95 | - | - | - | - | - | - |
| ***PF11_0209*** | all stages | 27.53 | 22.58 | - | - | - | - | - | - | - | - |
