# Supplementary material for: Inferring Developmental Stage Composition from Gene Expression in Human Malaria
Source: PLoS Comput Biol. 2013 Dec 12;9(12):e1003392. doi: 10.1371/journal.pcbi.1003392 (PMC3861035; doi:10.1371/journal.pcbi.1003392)
Supplement: Table S6 — Primer and probe sequences used in qRT-PCR. Sequences for the reverse and forward primers and minor groove-binding fluorescent probes used in the qRT-PCR assay. (DOCX) [file pcbi.1003392.s008.docx]

**Primer and probe sequences**

*PFE0065w* (ring)

Reverse: TTGCTAGGTAATATCCTTTTCTTTTTCC

Forward: GCAAAACAAGCCGTACATGTTG

Probe: 6FAM - TTG TTC ATC AAC TTT TAC AAC TT - MGBNFQ

*PF10_0020* (troph/schizont)

Reverse: GACGTTTGATTTGTTTCCTGTTTTATC

Forward: GGAATGATTTATTTGTTAATTAAAGATGTTG

Probe: 6FAM - ACG AGG AAA TTA GCT GAA GC - MGBNFQ

*PF14_0748* (early-mid gametocytes)

Reverse: TTGGCCACACTGCTCTAGGA

Forward: CTTATGTGCTGAATTTTGTGTTATGGT

Probe: VIC - CAC ATA ATG AAT TCA AGG GTA G - MGBNFQ

*PF14_0367* (mid-late gametocytes)

Reverse: TCCCTGTGTTTTTGCTCATCTTC

Forward: GTTACATTTCGACCCAGCATAAATT

Probe: VIC - CAG TGC ATA TTG TTG CCT GT - MGBNFQ

*PF11_0209* (all stages)

Reverse: CATAATGCTACTAACTACTAATATGCAAAAATATACC

Forward: CGCTAGAATTACATGGAGACAAATCA

Probe: VIC - AAA AGG TCA AGC CTT CAT T - MGBNFQ
